# Trust in the Healthcare System and Physicians in Croatia: A Survey of the General Population

**DOI:** 10.3390/ijerph19020993

**Published:** 2022-01-16

**Authors:** Krunoslav Nikodem, Marko Ćurković, Ana Borovečki

**Affiliations:** 1 Department of Sociology, Faculty of Humanities and Social Sciences, University of Zagreb, Ivana Lučića 3, 10000 Zagreb, Croatia; knikodem@ffzg.hr; 2 School of Medicine, University Psychiatric Hospital Vrapče, University of Zagreb, Bolnička Cesta 32, 10090 Zagreb, Croatia; markocurak@gmail.com; 3 Andrija Stampar School of Public Health, School of Medicine, University of Zagreb, Rockefellerova 4, 10000 Zagreb, Croatia

**Keywords:** trust, public, healthcare system, physicians, predictors, Croatia

## Abstract

Trust in healthcare systems and physicians is considered important for the delivery of good healthcare. A cross-sectional survey was conducted on a random three-stage sample of the general population of Croatia (N = 1230), stratified by regions. Of respondents, 58.7% displayed a high or very high level of trust in the healthcare system, 65.6% in physicians, and 78.3% in their family physician. Respondents’ views regarding patients’ roles in the discussion of treatment options, confidence in physicians’ expertise, and underlying motives of physicians were mixed. Respondents with a lower level of education, those with low monthly incomes, and those from smaller settlements had lower levels of trust in physicians and the healthcare system. Trust in other institutions, religiosity and religious beliefs, tolerance of personal choice, and experience of caring for the seriously ill and dying were predictors of trust in healthcare and physicians. Our findings suggest that levels of healthcare-related trust in Croatia are increasing in comparison with previous research, but need improvement. Levels of trust are lowest in populations that are most vulnerable and most in need of care and protection.

## 1. Introduction

Trust in the healthcare system and physicians is a considered key component of the delivery of good healthcare and achieving desirable health outcomes [1,2,3,4]. Trust is an optimistic acceptance of one owns vulnerable condition with a corresponding belief that one’s interests will be taken care of by physicians (interpersonal trust) or healthcare institutions or the system itself (impersonal, institutional trust) [5,6]. Although trust can correspond to trustworthiness, sometimes it does not. For example, patients can trust physicians and institutions that are not deserving of their trust. They can also fail to trust those that are deserving of their trust [5]. Trust is not the same as satisfaction with healthcare services, which is often used to evaluate the quality of healthcare. Satisfaction is an assessment of service delivery, while trust is an attitude formed around certain characteristics of those that deliver care and the dimensions of one’s relationship with them that take place within the healthcare system as a whole [5]. Inasmuch, potential determinants of trust can be personal (physicians or healthcare providers in general or a specific physicians or care provider) or institutional (healthcare institutions, the healthcare system as a whole, or a specific institution or a part of healthcare system) [1,5,7]. Trust in healthcare has multiple dimensions. In the literature, the following dimensions are usually associated with trust: fidelity (keeping patients best interest in mind and not taking advantage of them), honesty (telling the truth and avoiding intentional dishonesty), confidentiality (protection and proper use of sensitive patients’ information), competence (a more technical dimension of trust which, opposed to previously mentioned relational ones, entails avoiding mistakes and producing the best achievable results), and general trust (stemming from implementation of all previously mentioned dimensions) [5,8]. 

Measuring trust in healthcare is not an easy or straightforward task. Many have put forward their preferred methodology for the measurement of trust in healthcare [9,10,11,12,13,14]. There are several predictors of trust in healthcare settings found in existing literature. They can be classified according to patients’ characteristics (demographic characteristic, patient personality, worldview), physicians’ characteristics (demographic and professional characteristic, personality, and behavior), and relational or situational factors (length of physician–patient relationship, possibility of selection of a physician, commodification of a healthcare system, insurance and reimbursement schemes, and organizational practices) [5]. Some of these have been shown to be strong predictors such as physicians’ personality traits and behaviors including communication styles and interpersonal skills, lack of continuity of care, types of health insurance schemes and whether they allow the selection of physicians by patients, and health literacy of patients. Some are considered to be weak predictors, such as the length of the physician–patient relationship, the demographic characteristics of physicians and patients, and patients’ personality traits [5].

The Croatian healthcare system has been in transition for many years and has undergone a number of reforms. For each of the implemented reforms, the objective was to optimize the healthcare system in line with the current government’s budget to achieve sustainability in the long run [15]. It is a healthcare system that is under the constant threat of further commercialization and commodification (the incentive to transform healthcare from a granted right into a commodity), and research has shown that healthcare commodification with the processes of deregulation and privatization may compromise the public’s general trust in physicians [16].

Croatia is the country with the lowest institutional trust and one of the lowest societal trusts in Europe. According to the results of the European Values Study for 2017, only 7.2% of Croatian citizens trust the parliament, 9.6% trust the government, and 13.6% trust the judiciary, while only 13.6% of Croatian citizens trust other people. Nonetheless, confidence in the healthcare system is still rather high, with 41.6% of citizens having confidence in the healthcare system [17,18] (Table 1). 

The reasons for this situation can be found in the negative legacy of the former communist system, widespread corruption in all areas of life and constant unsubstantial “reforms” without clear goals [17,19].

In the Republic of Croatia, no systematic research has been done so far on the trust of the general public in the healthcare system and physicians. Thus, the primary aim of this study was to examine the trust in physicians and the healthcare system in Croatia and to investigate possible predictors of trust in physicians and the healthcare system. 

## 2. Materials and Methods

### 2.1. Design

A cross-sectional omnibus survey was conducted on a random three-stage sample of the general population, stratified by regions, the respective counties, and locations within those counties in the Republic of Croatia. The survey was a part of the larger research project Values and Decisions at the End of Life (VAL-DE-END). The aim of the project is to make a multi-stepped, multi-sourced, and comprehensive analysis of end-of-life issues in the Republic of Croatia. So far, we have published our analysis regarding the general public’s attitudes towards death and dying [20]. The research on trust of general populations in physicians and healthcare is also part of the project agenda. 

### 2.2. Sample

The sample (N = 1203) of adult citizens (18 years and older) from the Republic of Croatia was constructed on the basis of the 2011 census, which was the last official census done in Croatia (Table 2). The number of respondents on the overall level allows inference of the target population with a maximum sample error of +/−2.8%. 

By including weights, the sample becomes nationally representative in terms of gender, age, education, and regional representation. The real shares of the regions were adjusted by the weighting process based on the 2011 census. 

Although the probabilistic sampling design seeks to ensure the representativeness of the sample within each region according to key sociodemographic variables (gender, age, and educational structure of respondents), the shares of respondents within the sample are not fully consistent with the corresponding reference population shares. In order to ensure the representativeness of the sample according to the mentioned selected sociodemographic variables, the data were subsequently weighted according to the key selected sociodemographic variables. The weighting procedure was performed using the Random Iterative Method (RIM method), a method that adjusts the sample structure to population parameters on several variables at the same time. The adjustment procedure is achieved with as little distortion of the variables that have undergone the weighting procedure as possible. Although according to the stratification sampling plan, the percentages of respondents within the realized sample are proportional to their territorial population parameters, and according to the criterion of territorial distribution, the realized sample is representative, given the smaller deviations of the territorial distribution of the realized sample from the population parameters, the sample was subsequently weighted by regional distribution. Subsequent weighting procedures realized the sample and harmonized it with the population parameters according to the selected characteristics of the sociodemographic structure; i.e., a representative sociodemographic structure of the sample was provided. In other words, after the weighting procedure, the shares of gender, age, and educational groups examined in the realized sample do not deviate by more than +/−1% from the share of gender, age, and educational groups in the population. The weighting procedure ensured that the shares of respondents in the sample are equal to the shares of the general population in terms of the relevant variables.

The sampling methods used in the research included stratified random sampling. All adult citizens 18 years of age and older were included in the sample. According to the proportional share of the population, the required number of respondents in each county was predefined. Settlements (cities, villages) were randomly selected, taking into account the rural–urban distribution in each county. The place of residence in each settlement was randomly selected by the random walk method, in which randomly selected surveyors entered every third household on their right-hand side. In each household, the last birthday method was used as a selection criterion. Therefore, random selection of the respondents together with the weighting procedure allowed the generalization of the obtained results to the population. 

After initial contact with a household by surveyors and the detection of potential respondents in the case of acceptance to participate in the survey, the surveyors explained to the participants the background of the study and the goals of the study. This methodology yielded a response rate of 30%. Replying to the questionnaire was voluntary and was not rewarded; the anonymity of the participants was ensured. The questionnaire was administered by trained surveyors on site. The survey was conducted during November and December, 2019, before the onset of the COVID-19 pandemic.

### 2.3. Instrument

The questionnaire was developed as a part of the VAL-DE-END project for the purpose of examining the attitudes of the population, which aimed to cover several themes (an omnibus questionnaire covering issues of attitudes towards death and dying, end-of-life decision-making and practices, trust in physicians and healthcare, and attitudes to other bioethical issues). 

The questionnaire was developed through several consecutive phases. Firstly, an extensive literature search was performed in order to identify and analyze existing questionnaires used for similar purposes. Items informed by the literature review were assessed by experts in survey methodology and experts from the respected field of study. All items that were originally available in English were translated twice from English to Croatian by independent translators, followed by translations back to English by two additional translators. The questionnaire was pre-tested on a small convenience sample of the target population (N = 50).

The final questionnaire has 90 items. However, for the purpose of this analysis, we have included 49 items from the questionnaire. In this analysis, we focus mainly on attitudes about trust in physicians and the healthcare system in Croatia. 

Of the 49 items, 10 items were related to sociodemographic characteristics of the sample, two items from European Values Study [21] measure religiosity (one measuring recent attendance of religious services and one item explicitly asking about participants’ religious beliefs), four items measure certain religious beliefs (belief in God, life after death, heaven and hell), and four items from the European Values Study measure justification of divorce, abortion, casual sexual intercourse, and homosexuality. An additional two items measure political orientation and tolerance of personal choice. Ten items measure experience of the death of family members and loved ones, and experiences of caring for the terminally ill and seriously ill.

Nine items from European Values Study measuring trust in different institutions and organizations (police, army, church, educational system, parliament, judiciary system, government, healthcare system, and other people) were used to measure general trust in different institutions and the healthcare system in general [21].

Eight items were used to measure trust in physicians and the healthcare system in Croatia. One item was used to measure trust in the healthcare system in Croatia as a whole and one item was used to measure general trust in physicians in Croatia. Six items measured specific determinants of trust in physicians. The selected determinants included: “autonomy engaging/enabling communication”, “mutual trust and respect”; “expertise” (as the technical element of competence), “carefulness and thoroughness” (as showing more concern, the interpersonally-related element of competence), and “integrity” or “reliability” (two items measuring physicians’ concern for patients’ needs as related to: (1) the physicians’ own well-being and (2) physicians’ earnings). These slightly modified items stem from the research and instruments constructed by previous studies [10,11,12,13,14,22].

### 2.4. Data Analysis

Descriptive statistics, including frequencies and percentages, were used to describe the baseline characteristics of the participants. The data were processed using IBM SPSS Statistics 26. In addition to descriptive statistics, we used the chi-square test to determine differences, and multiple regression analysis. 

## 3. Results

### 3.1. Sociodemographic Characteristics of Respondents

One thousand, two hundred and three respondents participated in the study. The average age of the respondents was 48.21 years. More than half of the respondents were female, 43% of the respondents were married, and slightly less than half of the respondents had either one child or two children. More than half of the respondents had some form of secondary education (vocational school, high school). Around two thirds of the respondents were employed, 43.3% of the respondents lived in settlements with fewer than 2000 inhabitants, and 35.3% of the respondents had incomes below the average income in the Republic of Croatia (Table 3). 

### 3.2. Respondents’ Religious Beliefs and Practices, Political Orientation, and Tolerance of Personal Choice

Of respondents, 64.9% considered themselves religious, 76% believe in God, 17.3% attended religious ceremonies once a week, 19.0% attended religious ceremonies only for religious holidays, and 19.7% never attended religious ceremonies.

Of the respondents, 10.8% considered themselves to be “left” on the political spectrum and 19.1% to be “right”; 17.5% considered themselves “liberal” and 10.2% “conservative”. 41.9% of the respondents thought that divorce could be justified, 40.3% of the respondents could not justify abortion, and 42.8.9% could not justify homosexuality.

The highest percentage of religious persons was found in Southern Croatia (74.0%) and the lowest in the Zagreb region (56.4%). Furthermore, Southern Croatia had the highest percentage of respondents who attend religious ceremonies per week (33.1%), while in Istria and the Primorje regions this percentage was the lowest in Croatia (7.4%).

The highest percentage of respondents who declared themselves as left-wing on the political spectrum was found in the Zagreb region (17.4%), The majority of respondents who declared themselves as right-wing could be found in Southern Croatia (37.5%), where those who consider themselves “conservative” were also the most numerous (19.7%). The highest percentage of respondents who declared themselves as liberal was found in Northern Croatia (26.2%).

The death of a close person had been experienced by 74% of respondents, 41.8% had experienced the death of a mother, 50.4% had experienced the death of a father, and 6.4% experienced the death of their own child. 42.2% of respondents had cared for a seriously ill person and 34.6% of respondents had cared for a terminally ill person.

### 3.3. Level of Trust in Institutions and Society 

A high or very high level of trust in both the educational system and healthcare system was displayed by 58.7% of respondents in Croatia, 56.5% of respondents displayed a high or very high level of trust in the military, 40.7% in the police, 21.1% in the church, 17.2% in the judiciary system, 15.6% in the parliament, and 12.3% in the government. The statement that majority of the people can be trusted was endorsed by 29.5% of the respondents.

The highest level of social trust was found in the Zagreb region (39.2%), and the lowest in Northern Croatia (22.1%).

### 3.4. Level of Trust in Physicians and Healthcare

The results show (Table 3) that 58.7% of respondents express confidence in the health system. The majority of respondents (65.6%) answered in the affirmative to the general statement that physicians in Croatia can be trusted, and an even higher percentage of respondents (78.3%) stated that they trusted their family physician.

The highest level of trust in the healthcare system was found in Northern Croatia (76.7%), and the lowest in Eastern Croatia (50.3%).

### 3.5. Specific Dimensions of Physician Patient Relationship

About 52% of respondents agreed and about 20% of respondents disagreed with the statements that described physicians in a positive manner, such as that physicians are generally extremely thorough and attentive. About 45% of respondents agreed with the statement that there is trust and mutual respect between physicians and patients in Croatia and 48% of respondents agreed with the statement that physicians discuss all treatment options with their patients. However, about 24% of respondents thought the opposite. 

An equal percentage of respondents agreed or disagreed (about 32%) with the statements that physicians sometimes care more about what is good for them than about the needs of their patient. About 32% of the respondents agreed and slightly more (about 36%) disagreed with the statement that the expertise of physicians in Croatia is not as good as it should be. Finally, only 7.7% agreed with the statement that physicians care more about their income than about their patients (Table 4).

The analysis of the chi-square test results regarding differences in respondents’ answers with respect to the basic sociodemographic characteristics of the respondents (gender, age, level of education, monthly income, and place of residence) is shown in Table 5.

Respondents from regional centers and Zagreb (the capital of Croatia), those with secondary education, and those with higher monthly incomes are more inclined to trust the healthcare system in Croatia, in contrast to respondents from smaller settlements (up to 2000 inhabitants), those with a lower education and those with lower income. Furthermore, respondents with a lower level of education, those with lower incomes, and those from smaller settlements are more inclined to believe that, generally speaking, physicians in Croatia cannot be trusted, while those with higher incomes and those from regional centers more often believe the opposite. Older respondents (65+) are more inclined to specifically trust their family physician, and those from smaller settlements are less inclined to do so.

Females, respondents with a higher level of education, those with higher incomes, and those from regional centers were more inclined to agree with positive statements about physicians and measured determinants of trust in physicians, as opposed to males, respondents aged 48 to 64, those with a low level of education and low incomes, and those from smaller settlements, who tend to agree with corresponding negative statements, for example, that physicians care more about their income than about their patients. However, respondents under the age of 30, those with high monthly incomes, and those from smaller settlements (up to 2000 residents) were more likely to agree with the statement that the expertise of physicians in Croatia is not as good as it should be.

Respondents with a secondary level of education, respondents with high incomes, and those from regional centers were more inclined to give a positive answer to the statement that physicians discuss all treatment options with their patients while older respondents, those with a low level of education, those with low incomes, and those from settlements up to 50,000 inhabitants tend to disagree with this statement. 

### 3.6. Multiple Regression Analysis

In the further analysis, we used multiple regression analysis to analyze one predictor set (28 items) containing questions about general and institutional trust, health status, the participants’ experiences of death, their religiosity, political orientation, and tolerance of personal choice. Each item was analyzed separately with respect to the predictor set and results are shown in Table 6.

The results show that the selected items of predictor set explain about 40% of the criterion in the case of general trust in the healthcare system. The most important predictors in that sense are institutional trust (in the educational system, the military, parliament, and the Church), disapproval of abortion, the experience of the death of a friend, and general trust. With regard to general trust in physicians, the most important predictors were institutional trust (in the educational system and in the police), religiosity, belief in hell, and the experience of grandparents’ deaths. The explanatory power of the criterion is about 22%. In the case of more specific trust, such as trust in a personal family physician, the explanation by predictors is about 18%, while the most important positive predictors are also institutional trust (in the educational system and the army), the experience of the death of a friend, and belief in hell. 

Regarding more specific determinants of trust in physicians, institutional trust (educational system and judiciary), belief in hell, disapproval of abortion, and right-wing political orientation are important predictors of the view that physicians are generally extremely thorough and careful. Similarly, institutional trust (educational system and judiciary), right-wing political orientation, belief in hell, the experience of a friend’s death, general trust, and disapproval of abortion are important predictors regarding the view that there is trust and mutual respect between physicians and patients. The predictor set explains 19% of the criterion. Similar results were obtained for the opinion that physicians discuss all treatment options with their patients, but with a slightly lower predictive power (15%). Respondents who have confidence in the education system, police, and judiciary; those who believe in the existence of hell, disapprove abortion, and have right-wing political orientations are more inclined to this opinion. However, one difference from the previous set is that those who do not trust other people are also more inclined to this opinion.

The selected predictor set predicts about 14% of the criterion variable in the case of the attitude that physicians sometimes care more about what is good for them than about the needs of their patients. Respondents who do not trust the educational system and the judiciary, those who have not had the experience of the death of a friend or their own child, who do not approve divorce, and are politically left-wing are more inclined towards this attitude. An even smaller predictive power of the variance of the criterion was obtained from the other two negative statements about measured determinants of trust in physicians, at 12.5% and only 10%, respectively. Respondents who are not religious, do not believe in hell, have not had the experience of the death of a close person (a friend); do not trust important institutions such as the education system, the police, and political parties; and are left-oriented are more likely to think that doctors care more about their income than about their patients. For the view that the expertise of doctors in Croatia is not as good as it should be, the most important predictors are distrust in the Church, non-religiosity, distrust in the judiciary, and lack of experience of caring for a seriously ill person.

## 4. Discussion

The aim of this research was to examine trust in physicians and the healthcare system in Croatia and to investigate the possible predictors of the trust in physicians and the healthcare system.

Our research shows higher levels of trust in healthcare (58.7%) than in previous research done in Croatia (in 1991, 46% of respondents trusted healthcare system, in 2008, 40%, and in 2018, 43%) [23]. This is higher than in a recent study in China, where only 28% of respondents trusted the healthcare system [24]. 

The order of trust in specific institutions has changed from previous research. In our research, the respondents had highest level of trust in the healthcare system and educational system, then in the military and police. In 1999 (just after the end of war in Croatia), the respondent displayed highs levels of trust in military, then in the educational system, then in the church, then in the police, and then in the healthcare system. In 2008, they trusted the educational system the most, then the church, then the military, then the healthcare system, then the police. In 2018, the respondents trusted the military the most, then the educational system, then the police, then the healthcare system, then the church [23]. 

In our research, the majority of respondents (65.6%) believed that physicians in Croatia can be trusted and an even higher percentage (78.3%) put trust in their family physician. These numbers are quite high and the respondents displayed higher levels of trust in physicians then in the healthcare system. This is not unexpected, since research has shown that despite problems in the healthcare system, the trust in healthcare practitioners remains very high [7,25]. Moreover, trust in healthcare practitioners is more connected to interpersonal familiarity with specific practitioners, in this case the family physicians [26,27]. 

When it comes to views on specific issues in the physician–patient relationship, our respondents had mixed opinions on them. More than 50% of the respondents did not discuss treatment options with their physicians in an adequate manner. Our data shows lower levels of adequate discussion of treatment procedures than the research done by Vučemilo et al. in hospitals in Croatia, which showed that 57% of patients assessed the obtained level of information about their treatment options as high, 37% as average, and only 5% as low [28].

In our research, 32% of respondents agreed with the statement that physicians sometimes care more about what is good for them than about the needs of their patient, which is higher than in the research done by Ćurković et al., where 17.8% of the respondents agreed with a similar statement that physicians always or almost always put their own interest in front of their patients’ health [29]. However, only 7.7% respondents in our study agreed with the statement that physicians care more about their income than about their patients. The respondents in our study also had had mixed views on the expertise of physicians in Croatia. 

Sociodemographic characteristics (age, level of education, income, and the type of settlement) in our research seem to be predictors of trust in the healthcare system and physicians in general, but also specific determinants of trust on interpersonal level. Although not always considered to be strong predictors or even predictors of trust at all, the aforementioned sociodemographic characteristics have some influence on general trust in physicians and the healthcare system, and on specific determinants of trust on the interpersonal level [5,12,30,31]. Research has also shown that persons with higher health literacy, which is often in connection with higher level of education, display higher levels of trust in physicians and the healthcare system [32]. Moreover, older persons are more inclined to trust physicians and in our case their family physicians [5].

The Croatian healthcare system has been under constant pressure of commodification [15]. This causes certain levels of inequality among different income groups. This is probably the reason why lower income groups found it difficult to place trust in the healthcare system and physicians in our study [15,33]. The same respondents had more negative views on physicians’ priorities and their primary interests, which is also an opinion shared by respondents in previous studies in Croatia [29]. 

Our research also shows that if you do not have at least a secondary level of education, you will not be able to comprehend the information about treatment options given to you by physicians. This is in accordance with previous research done in Croatia by Vučemilo et al. on informed consent forms, which indicated that patients needed a college education to understand informed consent forms used in everyday clinical practice in Croatia [34]. 

Another issue emerging from our study is that people who do not live in big cities—this being quite a high percentage of our population—displayed lower levels of trust in the healthcare system and physicians in general, even their own family physicians. In our opinion, this may be influenced by the distribution of healthcare resources and the accessibility of healthcare. In Croatia, the geographical distribution of healthcare infrastructure and human resources is uneven. The largest number of hospitals and health workers located in central Croatia and in big cities. Croatia faces a shortage of physicians and nurses, particularly in rural areas [35]. According to the Euro Health Consumer Index (EHCI), which measures healthcare quality and accessibility, Croatia was ranked 24th out of 35 European countries in 2018 [36]. Research has shown that income and accessibility of healthcare play an important role when it comes to trust in one’s physician [33,37]. However, respondents under the age of 30, those with high monthly incomes, and those from smaller towns were more likely to agree with the statement that the expertise of physicians in Croatia is not as good as it should be. Reasons for this need to be further investigated.

The most important predictors of trust in our research were institutional trust, religiosity, political orientation, tolerance of personal choice, and previous experiences with death and caring for seriously ill persons. Research has shown that there is a link between vertical trust (the trust that people place in higher authorities) and trust in the healthcare system in general and in healthcare practitioners [38]. Levels of public trust in healthcare are affected by spill-over effects from high or low levels of public trust in other parts of the society. Many actors inside and outside the healthcare system can influence public trust in healthcare system [39]. The ideology can be an indicator of trust in government and medical experts, which in turn helps to explain individual-level variation with regards to attitudes about certain healthcare issues like vaccination [40]. Finally, those who have encountered death and serious illness put more trust in physicians and healthcare, since patients’ trust in physicians is conditional and is earned by their experience of care and the nature of their relationship with their physician [41].

Our research was done prior to the COVID-19 pandemic. Croatia is currently struggling with low vaccination rates below 60% of the adult population, one of the lowest in the EU. There is a surge of new COVID-19 cases and mandatory COVID-19 passes have bene introduced for access to all governmental institutions, with ongoing discussion of further expansion of their mandatory use. This measure has provoked wide and relatively massive protests that occur almost on daily basis. This seems to create further polarization of society and further fosters lack of social cohesion, already previously observed in studies done in Croatia within COVID-19 context [42]. Recent studies of trust in healthcare in Croatia during COVID-19 pandemic shows levels of trust below the average in EU (5.7 on the scale of 10, while the EU average is 6.4), but higher levels than in Hungary, Poland, and Slovakia [43]. Further research is needed to investigate possible impact of the COVID-19 pandemic on levels of trust in physicians and healthcare in Croatia. 

## 5. Conclusions

Our research shows that the level of trust in healthcare in Croatia is increasing in comparison to previous research and that trust in healthcare is connected with the level of trust that people put into other social structures. However, respondents’ views regarding specific dimensions of the physician–patient relationship (a patient’s role in discussion of treatment options and confidence in physicians’ expertise and motives) are not satisfactory. Moreover, sociodemographic characteristic, religiosity, political orientation, tolerance of personal choice, and interpersonal experiences between physicians and patients influence trust in the healthcare system and physicians. Levels of trust are lowest in populations that are most vulnerable, arguably those that are most in need of care and protection. Therefore, the current situation regarding trust in the healthcare system and physicians needs improvement. 

## 6. Limitations

The response rate was 30% which is low, but this is more or less a usual rate for similar studies with the general public in Croatian society; for example, the response rate for European Values Study field work in 2017/2018 was 38%. Furthermore, the income data are self-reported, which limits their reliability. In the case of post-communist countries, experts from the World Bank pointed out in 2003 that a considerable part of income—for various reasons—tends to be concealed. Our research was done prior to the COVID-19 pandemic. The possible impact of the COVID-19 pandemic on trust in healthcare and physicians in Croatia needs to be investigated.

## Figures and Tables

**Table 1:** Societal trust and trust in important institutions in Europe 2017/2018 (in percentage, %).

Countires	Societal Trust	Parliament	Government	Judiciary	Health Care System
*Developed Democractic Countries*					
Denmark	73.9	46.3	39.1	80.2	75.5
Finland	68.4	44.7	41.6	78.9	82.6
France	26.3	33.1	30.7	58.3	83.6
Germany	42.6	38.5	34.7	61.9	64.1
Great Britain	40.2	32.4	29.3	63.4	83.0
Netherlands	58.5	41.9	46.9	54.6	72.7
Norway	72.1	69.6	59.2	85.9	88.4
Sweden	62.8	63.3	50.7	76.1	78.4
Switzerland	58.5	57.6	66.3	70.0	70.5
*Post-communist Countries*					
Bosna and Herzegovina	9.6	16.2	18.3	27.9	52.0
Bulgaria	17.1	15.0	19.9	14.5	24.8
Croatia	13.6	7.2	9.6	13.6	41.6
Czech Republic	21.1	13.3	17.6	37.2	60.8
Hungary	27.2	34.8	37.6	48.4	37.6
Monte Negro	21.7	33.2	36.1	34.6	40.5
North Macedonia	15.1	31.7	25.7	33.8	46.5
Poland	24.1	19.5	23.1	35.0	45.3
Romania	12.7	16.9	17.9	44.1	44.3
Serbia	16.3	20.6	28.7	29.1	40.9
Slovakia	21.4	39.0	30.4	33.9	54.7
Slovenia	25.3	15.0	14.1	22.1	44.9

Source: EVS Data Set 2017/2018.

**Table 2:** Total number and percentage of adult citizens of the Republic of Croatia according to the 2011 census and the achieved sample within each region.

Region	Number of Inhabitants in the Age Group 18+ in the Population	Percentage of Respondents in the 18+ Age Group in the Population	Number of Surveyed Members of the Age Group 18+ in the Sample	Percentage Share of Surveyed Members of the Age Group 18+ in the Sample
Zagreb and its surroundings	904,960	25.95%	300	25.00%
Northern Croatia	531,875	15.25%	190	15.83%
Eastern Croatia	643,688	18.46%	230	19.17%
Central Croatia	292,065	8.38%	100	8.33%
Istria and Primorje Regions	425,534	12.20%	150	12.50%
Southern Croatia	688,912	19.76%	230	19.17%

**Table 3:** Sociodemographic characteristics of respondents (N = 1203).

Sample Characteristics	N (%)
**Gender**	
Male	572 (47.6)
Female	631 (52.4)
**Marital status**	
Married	517 (43)
Not married	279 (23.2)
Divorced	145 (12.1)
Widowed	159 (13.2)
Extramarital union	77 (6.4)
**Number of children**	
Childless	389 (32.2)
One child	240 (20)
Two children	343 (28.5)
Three children	134 (11.2)
Four children	70 (5.9)
Five to seven children	17 (1.4)
**Education**	
Unfinished primary school	79 (6.6)
Primary school (8 years)	257 (21.4)
Secondary vocational (1–3 years)	239 (19.9)
Secondary vocational (4 years and longer)	318 (26.4)
High school	103 (8.6)
2–3 years of higher education	69 (5.7)
College	110 (9.1)
Master’s degree	23 (1.9)
PhD degree	4 (0.3)
**Employment**	
Employed	789 (65.6)
Unemployed	28 (2.3)
Retired	245 (20.4)
**Type of settlement**	
less than 2000 inhabitants	521 (43.3)
between 2–10,000 inhabitants	191 (15.9)
between 10–50,000 inhabitants	152 (12.6)
between 50–100,000 inhabitants	60 (5)
between 100–500,000 inhabitants	121 (10.1)
with more than 500,000 inhabitants	158 (13.1)
Income per household *	
less than 5512.50 HRK	424 (35.3)
5512.50–11,025.00 HRK	372 (30.9)
11,025.00–22,050.00 HRK	224 (18.7)
22,050.00 HRK and more	27 (2.3)

* The average net salary in November 2020 was HRK 6823, and the minimum salary for 2021 is HRK 3400 net.

**Table 4:** Distribution of respondents ’ answers.

Questions and Answers	Number (%)
1. Trust in the healthcare system.	
Non-existent	109 (9.1)
Not at all significant	349 (29.0)
Do not know	34 (2.8)
Significant	516 (42.9)
Very significant	194 (16.2)
2. Physicians in Croatia can be trusted.	
Not at all	17 (1.5)
Mostly not	147 (12.3)
I do not know	249 (20.7)
Mostly yes	655 (54.4)
Yes, completely	134 (11.2)
3. I have confidence in my family medicine physician (general practitioner).	
Not at all	13 (1.1)
Mostly not	86 (7.2)
I do not know	161 (13.4)
Mostly yes	624 (51.9)
Yes, completely	318 (26.4)
4. Physicians are generally extremely thorough and attentive.	
Not at all	52 (4.3)
Mostly not	183 (15.2)
I do not know	345 (28.7)
Mostly yes	536 (44.6)
Yes completely	87(7.3)
5. There is trust and mutual respect between physicians and patients in Croatia.	
Not at all	50 (4.1)
Mostly not	205 (17.1)
I do not know	410 (34.1)
Mostly yes	411 (34.2)
Yes, completely	126 (10.5)
6. Physicians discuss all treatment options with their patients	
Not at all	93 (7.7)
Mostly not	192 (16.0)
I do not know	347 (28.8)
Mostly yes	409 (34.0)
Yes, completely	162 (13.5)
7. Physicians sometimes care more about what is good for them than about the needs of their patients.	
Not at all	90 (7.5)
Mostly not	296 (24.6)
I do not know	440 (36.6)
Mostly yes	315 (26.2)
Yes, completely	62 (5.2)
8. The expertise of physicians in Croatia is not as good as it should be.	
Not at all	140 (11.6)
Mostly not	287 (23.9)
I do not know	386 (32.1)
Mostly yes	312 (25.9)
Yes, completely	78 (6.5)
9. Physicians care more about their income than about their patients.	
Not at all	115 (9.6)
Mostly not	276 (22.9)
I do not know	428 (35.6)
Mostly yes	291 (24.2)
Yes, completely	92 (7.7)

**Table 5:** Differences in respondents’ answers according to sex, age, education, monthly income, and size of place of residence (chi-square test results).

Statements and Answers	Sex		Age				Education			Income, Kuna per Month				Size of Place of Residence				
	Male	Female	65	64–48	47–31	30	Elementary	High	College and Higher	To 5512.50	5512.50–11,025.00	11,025.00–22,050.00	22,050.00 and More	<2000	2001–10,000	10,001–50,000	50,001–500,000	500,001<
1. Trust in the healthcare system.																		
None or weak	39.7	36.7	43.9	34.8	35.5	40.2	45.4	34.7	37.4	45.3	32.8	21.4	33.3	43.2	38.7	40.4	29.1	29.1
Don’t know	2.4	3.2	3.5	2.2	2.7	3.6	2.4	3.3	1.9	3.1	2.2	0.9	7.4	2.1	2.6	7.3	2.2	1.9
High and very high	57.9	60.2	52.5	63.0	61.8	56.2	52.2	62.0	60.7	51.7	65.1	77.7	59.3	54.7	58.6	52.3	68.7	69.0
Chi-square		1.544			9.137			11.858				47.366				31.616		
df		2			6			4				6				8		
*p*		0.462			0.166			<0.02				<0.001				<0.001		
2. Physicians in Croatia can be trusted.																		
Disagree	13.8	13.6	15.3	12.4	14.3	13.2	19.0	12.1	10.1	18.0	14.5	6.3	7.1	16.3	13.1	9.9	9.9	13.9
Neither	22.9	18.7	14.5	19.6	23.0	25.6	16.7	22.1	22.7	21.0	19.8	23.2	7.1	20.6	25.1	27.6	16.0	14.6
Agree	63.3	67.7	70.2	68.0	62.7	61.2	64.3	65.8	67.1	61.0	65.7	70.5	85.7	63.1	61.8	62.5	74.0	71.5
Chi-square		3.445			12.081			13.966				22.697				20.285		
df		2			6			4				6				8		
*p*		0.179			0.060			<0.001				<0.001				<0.001		
3. I have confidence in my family medicine physician (general practitioner).																		
Disagree	9.8	7.0	7.1	8.8	8.1	9.1	8.0	8.5	7.8	9.5	8.6	5.8	3.6	11.3	7.8	7.9	3.9	4.4
Neither	14.0	13.0	7.5	9.4	17.9	19.0	10.1	13.8	17.5	11.8	15.1	15.0	7.1	13.6	12.5	13.8	16.0	10.8
Agree	76.3	80.0	85.5	81.8	74.0	71.8	81.8	77.7	74.8	78.7	76.3	79.2	89.3	75.0	79.7	78.3	80.1	84.8
Chi-square		3.558			27.402			6.346				6.696				16.532		
df		2			6			4				6				8		
*p*		0.166			<0.001			0.175				0.350				<0.04		
4. Physicians sometimes care more about what is good for them than about the needs of their patients.																		
Disagree	27.3	36.3	35.0	32.9	32.2	27.4	29.7	32.6	34.5	29.8	36.2	33.6	29.6	30.5	27.2	34.2	34.6	38.0
Neither	40.2	33.4	35.8	32.9	40.0	38.5	29.1	39.8	38.3	33.3	36.5	42.0	55.6	32.2	39.3	32.9	46.7	39.2
Agree	32.5	30.3	29.1	34.3	27.8	34.1	41.2	27.6	27.2	36.9	27.3	24.3	14.8	37.2	33.5	32.9	18.7	22.8
Chi-square		11.861			8.589			22.938				20.178				32.266		
df		2			6			4				6				8		
*p*		<0.01			0.198			<0.001				<0.01				<0.001		
5. Physicians are generally extremely thorough and attentive.																		
Disagree	18.4	20.5	22.0	18.8	20.9	16.3	22.0	17.7	21.3	24.5	18.0	16.9	10.7	22.1	22.0	16.6	9.9	21.5
Neither	31.5	26.2	23.9	28.5	29.6	32.9	25.3	29.7	30.9	27.4	27.2	27.6	25.0	26.5	34.0	31.1	27.6	28.5
Agree	50.2	53.3	54.1	52.8	49.6	50.8	52.7	52.6	47.8	48.1	54.8	55.6	64.3	51.4	44.0	52.3	62.4	50.0
Chi-square		4.179			6.964			5.181				10.424				21.356		
df		2			6			4				6				8		
*p*		0.124			0.324			0.269				0.108				<0.01		
6. Physicians care more about their income than about their patients.																		
Disagree	28.3	36.3	33.7	33.4	34.3	27.5	31.5	31.5	37.4	31.8	33.9	36.0	40.7	27.3	32.1	30.9	46.4	36.1
Neither	37.9	33.6	34.1	33.1	37.9	37.5	24.9	41.1	35.9	31.1	37.4	35.1	51.9	36.9	34.2	37.5	29.3	38.6
Agree	33.9	30.1	32.2	33.4	27.8	35.1	43.6	27.4	26.7	37.0	28.8	28.9	7.4	35.9	33.7	31.6	24.3	25.3
Chi-square		8.807			6.861			38.551				16.232				27.056		
df		2			3			4				6				8		
*p*		<0.02			0.334			<0.001				<0.02				<0.001		
7. The expertise of physicians in Croatia is not as good as it should be.																		
Disagree	38.1	33.1	34.3	42.8	36.8	24.7	35.6	35.2	36.4	39.2	32.9	40.7	25.9	27.3	42.4	41.4	37.4	46.8
Neither	32.2	32.0	33.5	27.6	31.7	37.5	33.5	31.9	30.1	31.9	29.6	31.4	22.2	36.9	30.4	35.5	28.0	19.0
Agree	29.7	34.9	32.3	29.6	31.4	37.8	30.9	32.8	33.5	28.8	37.5	27.9	51.9	35.8	27.2	23.0	34.6	34.2
Chi-square		4.557			21.924			0.899				14.218				41.660		
df		2			6			4				6				8		
*p*		0.102			<0.001			0.925				<0.03				<0.001		
8. There is trust and mutual respect between physicians and patients in Croatia.																		
Disagree	24.6	18.1	20.8	25.4	19.4	17.9	28.2	18.9	17.0	25.9	18.5	13.3	14.3	24.6	25.8	23.0	8.2	17.8
Neither	33.7	34.5	37.3	26.8	37.0	37.8	33.2	33.6	37.4	34.0	31.6	39.6	17.9	37.3	36.8	34.9	29.1	23.8
Agree	41.7	47.4	42.0	47.8	43.6	44.2	38.6	47.5	45.6	40.1	49.9	47.1	67.9	38.1	37.4	42.1	62.6	57.3
Chi-square		8.296			14.401			15.938				25.366				53.812		
df		2			6			4				6				8		
*p*		<0.02			<0.03			<0.01				<0.001				<0.001		
9. Physicians discuss all treatment options with their patients																		
Disagree	21.5	25.7	33.5	25.3	20.1	16.3	31.8	20.0	22.3	27.7	23.4	18.7	7.4	28.8	18.3	27.8	10.4	24.2
Neither	33.0	25.0	25.6	25.6	31.1	33.3	22.8	29.8	35.0	27.2	26.6	32.4	25.9	27.8	39.8	29.1	23.6	24.2
Agree	45.5	49.3	40.9	49.0	48.8	50.4	45.4	50.2	42.7	45.2	50.0	48.9	66.7	43.4	41.9	43.0	65.9	51.6
Chi-square		9.705			26.037			22.587				13.218				51.029		
df		2			6			4				6				8		
*p*		<0.01			<0.001			<0.001				<0.04				<0.001		

The original 5-point scales where 1 = strongly disagree and 5 = strongly agree were recoded to 3 degrees, so that 1.2 = 1; 3 = 2, and 4.5 = 3. In Table 3, first the negative responses are presented, then the indecisive responses, and then the positive responses.

**Table 6:** Results of multiple regression analysis—predictors of trust in healthcare, physicians, and specific dimensions of the physician–patient relationship.

Trust Items	R²	Predictors	Standardised Coefficients	*p*
			beta	
1. Trust in the healthcare system.	0.404	Trust in the educational system	0.437	<0.001
		Trust in the military	0.130	<0.001
		Trust in Parliament	0.121	<0.001
		Trust in the Church	0.087	<0.030
		Abortion	−0.187	<0.001
		The experience of the death of a friend	0.141	<0.001
		Trust in other people	0.089	<0.008
2. Physicians in Croatia can be trusted.	0.218	Trust in the educational system	0.202	<0.001
		Trust in the police	0.196	<0.001
		Religiosity	0.170	<0.001
		Belief in hell	0.167	<0.003
		The experience of the death of a grandparent	0.142	<0.001
3. I have confidence in my family medicine physician (general practitioner).	0.176	Trust in the educational system	0.231	<0.001
		The experience of the death of a friend	0.162	<0.001
		Belief in hell	0.467	<0.001
		Trust in the military	0.110	<0.008
4. Physicians sometimes care more about what is good for them than about the needs of their patients.	0.137	Trust in the educational system	−0.137	<0.002
		The experience of the death of a friend	−0.158	<0.001
		Trust in the judiciary	−0.121	<0.005
		Divorce	−0.199	<0.001
		Political orientations	−0.105	<0.020
		The experience of the death of one’s own child	−0.103	<0.020
5. Physicians are generally extremely thorough and attentive.	0.183	Trust in the educational system	0.215	<0.001
		Belief in hell	0.359	<0.001
		Trust in the judiciary	0.143	<0.001
		Abortion	−0.171	<0.002
		Political orientations	0.110	<0.020
6. Physicians care more about their income than about their patients.	0.125	Religiosity	−0.202	<0.001
		The experience of the death of a friend	−0.142	<0.001
		Trust in the police	−0.097	<0.030
		Belief in hell	−0.143	<0.006
		Political orientations	−0.131	<0.005
		Trust in the educational system	−0.099	<0.030
7. The expertise of physicians in Croatia is not as good as it should be.	0.100	Trust in the Church	−0.109	<0.030
		Trust in the judiciary	−0.247	<0.001
		The experience of caring for a seriously ill person	−0.128	<0.002
		Religiosity	−0.113	<0.020
8. There is trust and mutual respect between physicians and patients in Croatia.	0.190	Trust in the educational system	0.220	<0.001
		Political orientations	0.155	<0.001
		Belief in hell	0.184	<0.001
		The experience of the death of a friend	0.171	<0.001
		Trust in other people	−0.120	<0.002
		Abortion	−0.176	<0.001
9. Physicians discuss all treatment options with their patients	0.150	Trust in the educational system	0.157	<0.001
		Trust in the police	0.112	<0.020
		Trust in the judiciary	0.104	<0.020
		Belief in hell	0.102	<0.020
		Abortion	−0.133	<0.008
		Trust in other people	−0.125	<0.002
		Political orientations	0.092	<0.040

## Data Availability

The data presented in this study are available on request from the corresponding author. The data are not publicly available due to privacy reasons.
